# Effects of *Lactobacillus acidophilus* on the growth performance and intestinal health of broilers challenged with *Clostridium perfringens*

**DOI:** 10.1186/s40104-018-0243-3

**Published:** 2018-03-27

**Authors:** Zhui Li, Weiwei Wang, Dan Liu, Yuming Guo

**Affiliations:** State Key Laboratory of Animal Nutrition, College of Animal Science and Technology, China Agricultural University, Beijing, 100193 People’s Republic of China

**Keywords:** Broiler, *Clostridium perfringens*, Intestine, *Lactobacillus acidophilus*, Necrotic enteritis

## Abstract

**Background:**

*Clostridium perfringens* is the main etiological agent of necrotic enteritis. Lactobacilli show beneficial effects on intestinal health in infectious disease, but the protective functions of lactobacilli in *C. perfringens*-infected chickens are scarcely described. This study examined the effects of *Lactobacillus acidophilus* (*L. acidophilus*) on the growth performance and intestinal health of broiler chickens challenged with *Clostridium perfringens* (*C. perfringens*) over a 28-day period. Using a 2 × 2 factorial arrangement of treatments, a total of 308 1-day-old male Arbor Acres broiler chicks were included to investigate the effects of *Lactobacillus acidophilus* (*L. acidophilus*) on the growth performance and intestinal health of broiler chickens challenged with *Clostridium perfringens* (*C. perfringens*) during a 28-day trial.

**Results:**

During infection (d 14–21), *C. perfringens* challenge decreased the average daily gain (*P* <  0.05), and increased feed conversion ratio and the mortality rate (*P* <  0.05). However, dietary supplementation with *L. acidophilus* increased the body weight of *C. perfringens*-infected broilers on d 21 (*P* <  0.05), and tended to decrease the mortality (*P* = 0.061). *C. perfringens* challenge decreased the villus height (*P* <  0.05), the ratio of villus height to crypt depth (*P* <  0.05) and *OCLN* (occludin) mRNA expression (*P* <  0.05), and increased the pro-inflammatory cytokine expression in the spleen and jejunum, the intestinal populations of *C. perfringens* and *Escherichia* (*P* < 0.05), and the serum content of endotoxin (*P* < 0.05), regardless of *L. acidophilus* supplementation. In contrast, dietary *L. acidophilus* reducedthe intestinal lesion score of challenged broilers (*P* < 0.05), the mRNA expression of pro-inflammatory cytokines, ileal populations of *Escherichia* and serum endotoxin content (*P* < 0.05), but increased the intestinal *Lactobacillus* populations (*P* < 0.05), irrespective of *C. perfringens* challenge.

**Conclusion:**

Dietary addition of *L. acidophilus* could improve the intestinal health and reduce the mortality of broilers suffering from necrotic enteritis.

## Background

Necrotic enteritis (NE) is a wide-spread poultry disease costing the global poultry industry approximately 2 billion U.S. dollars each year [1]. *Clostridium perfringens* (*C. perfringens*) type A is the main etiological agent of NE, and causes two forms of disease: a clinical form, with a sudden increase in flock mortality, often without premonitory signs, and a subclinical form, causing sub-optimal growth performance [2]. In-feed antibiotic growth promoters have been used to control NE; however, many countries forbid the dietary use of antimicrobials because of public concern over the emergence of antibiotic-resistant bacteria [3]. Outbreaks of NE have been increasing due to the withdrawal of in-feed antibiotics [4], indicating a threat to human food security. In recent years, probiotics have been used as an alternative to antibiotics. As probiotics, *Lactobicillus* species have been used to improve the intestinal health and growth performance of poultry. Lactobacilli produce bacteriostatic bacteriocin-like compounds [5] as well as acids, such as lactic acid, which decreases the pH of the gut. Competitive exclusion and antagonism have also been proposed as mechanisms by which lactobacilli species prevent the proliferation of pathogenic bacteria and regulate the intestinal flora [6–8]. Previous studies have shown that *Lactobacillus acidophilus* (*L. acidophilus*) can inhibit the pathogens [9, 10] and modulate the immunity [11]. However, it is unclear whether dietary *L. acidophilus* supplementation could improve the intestinal health and growth performance of broilers in a NE model. Therefore, we conducted a NE model-based feeding trial to investigate the effects of *L. acidophilus* on the intestinal permeability andhistomorphology, cytokine mRNA expression, and microbiota of broiler chickens challenged with *C. perfringens*.

## Methods

### Animals and experimental design

A total of 308 1-day-old male Arbor Acres broilers were used to study the effects of additives (without/with *L. acidophilus*), pathogen challenge (without/with *C. perfringens* challenge), and their interactive effects. A 2 × 2 factorial arrangement was used, with a completely randomized experimental design. All newly hatched healthy birds were weighed and randomly assigned into one of four treatment groups, with seven replicates in each group and 11 birds per replicate. The four groups consisted ofan untreated control (CTL), a *L. acidophilus* only supplementation group (LA), a *C. perfringens* only challenge group (CLG), and a *C. perfringens* challenge group supplemented with *L. acidophilus* (CLG + LA). All birds were housed in the cages (100 cm × 100 cm), and were offered free access to the feed and water throughout the 28-day trial.

### Diets and *L. acidophilus* supplementation

A corn-soybean-meal basal diet in mash form was formulated to meet the nutrient requirements of chickens, as per the recommended feeding standards for broilers in China (NY/T 2004) (Table 1). The ingredients of basal diet were mixed in the same batch to ensure that the experimental diets were identical in composition. A probiotic formulation of *L. acidophilus* LAP5 (Synlac Material Technology Co., Nanjing, China) was added to the basal diet at 40 mg/kg, providing 4.0 × 10^6^ cfu/kg of diet. Firstly, we calculated the exact amount of bacteria and basal diet that were needed to produce the experimental diets; secondly, the bacteria were diluted with a small amount of basal diet; thirdly, it would be mixed with the remaining basal diet using the shovel. The experimental diets were produced manually every 4 d.

**Table 1:** Composition of the basal diet (as-fed basis)

Item (%, unless otherwise indicated)	Composition
Ingredients	
Corn	51.38
Soybean oil	3.75
Soybean meal	40.71
CaHPO_3_·2H_2_O	1.86
Limestone	1.24
NaCl	0.35
* DL*-Met	0.20
Vitamine premix^a^	0.03
Trace mineral premix^b^	0.20
50% Choline chloride	0.25
Antioxidant	0.03
Total	100
Nutrient level^c^	
ME, MJ/kg	12.31
Crude protein	22.00
Lys	1.21
Met	0.52
Ca	1.00
Available phosphorus	0.45

^a^Vitamin premix (1 kg) contained: vitamin A, 50 MIU; vitamin D_3_, 12 MIU; vitamin K_3_, 10 g; vitamin B_1_, 10 g; vitamin B_2_, 32 g; vitamin B_12_, 0.1 g; vitamin E, 0.2 MIU; biotin, 0.5 g; folic acid, 5 g; pantothenic acid, 50 g; niacin, 150 g

^b^Trace mineral premix (1 kg) contained: copper, 4 g; zinc, 90 g; iron, 38 g; manganese, 46.48 g; selenium, 0.1 g; iodine, 0.16 g; cobalt, 0.25 g

^c^Calculated value based on the analyzed data for the experimental diets

### *C. perfringens* challenge

The *C. perfringens* challenge method used in this study was developed by Dahiya et al. [12], and modified by Liu et al. [13]. Briefly, a chicken *C. perfringens* type A field strain (CVCC 2030), originally isolated from a clinical case of NE, was obtained from the China Veterinary Culture Collection Center (Beijing, China). *C. perfringens* was cultured anaerobically on tryptose-sulfite-cycloserine agar for 18 h at 37 °C, and then aseptically inoculated into cooked meat medium and incubated anaerobically for 8 h at 37 °C. Birds in the challenge groups were orally gavaged once daily with actively growing *C. perfringens* (2.0 × 10^8^ cfu/mL, 1.0 mL per bird) during d 14–20, while the non-challenged birds were gavaged with the same volume of sterilized cooked meat medium.

### Sample collection

On d 21, one bird in each replicate was randomly selected and humanly euthanized to collect the blood, fresh digesta in the ileum (defined as the region between Meckel’s diverticulum and 2 cm cranial to the ileo-caecal junction) and cecum, spleen, and segments in the middle of jejunum (defined as the region from the end of duodenum to the Meckel’s diverticulum). The blood was collected aseptically from the wing vein into normal vacutainers. Serum was then obtained from the blood samples and stored at − 20 °C. Spleen and intestinal segments for mRNA isolation were frozen in liquid nitrogen. Fresh ileal digesta (collected from the segments between ileum midpoint and 2 cm proximal to the ileocecal junction) and cecal digesta were collected aseptically and stored at − 70 °C to determine the quantitation of the *C. perfringens*, *Escherichia* and *Lactobacillus* species populations.

### Growth performance

Body weight (BW) and feed intake (FI) for each replicate were measured on d 14, 21 and 28. Average daily gain (ADG), average daily feed intake (ADFI), and the feed conversion ratio (FCR) were calculated during the different periods (d 1–14, d 14–21 and d 21–28).

### Intestinal lesion score

The small intestine of each bird was excised and subjected to scoring for NE lesions on a scale from 0 to 4, as described by Liu et al. [13]. Briefly, 0 = normal intestinal appearance; 0.5 = severely congested serosa and mesentery engorged with blood; 1 = thin-walled and friable intestines with small red petechiae; 2 = focal necrosis, grey appearance and small amounts of gas production; 3 = sizable patches of necrosis, gas-filled intestine, and small flecks of blood; and 4 = severe extensive necrosis, marked hemorrhage, and large amounts of gas in the intestine.

### Intestinal histomorphology

A segment of ileum from each bird was fixed in 4% paraformaldehyde immediately after sacrifice and then embedded in paraffin. Transverse 5-μm sections were stained with hematoxylin and eosin, and then histomorphometrically examined using an Olympus optical microscope and ProgRes CapturePro software (version 2.7; Jenoptik, Jena, Germany). Ten villi were measured in each section and only complete and vertically oriented villi were measured. Villus height was measured from the tip of the villus to the crypt opening and the associate crypt depth was measured from the base of the crypt to the level of the crypt opening. Then the average values of villus height and crypt depth in each section were calculated, respectively. The ratio of villus height to relative crypt depth was calculated from these measurements.

### Serum endotoxin content

The barrier integrity and function were evaluated using an indirect method by measuring the serum endotoxin levels. A Quantitative Chromogenic End-point Tachypleus Amebocyte Lysate Endotoxin Detection Kit was used, following the manufacturer’s instructions (Xiamen TAL Experimental Plant Co., Fujian, China).

### Gene expression analysis

Total RNA isolation was carried out by using Trizol reagent (Invitrogen Life Technologies, Carlsbad, CA) according to the manufacturer’s instructions. The concentration and purity of total RNA was monitored using a Nanodrop ND-1000 spectrophotometer (Thermo Fisher Scientific Inc., Waltham, MA, USA). One microgram of total RNA was reverse transcribed by a reverse transcription kit (Takara Bio Inc.) according to the manufacturer’s instructions. Reverse transcription was performed at 42 °C for 2 min, 37 °C for 15 min, followed by heat inactivation for 5 s at 85 °C. All of the cDNA preparations were stored frozen at 20 °C until further use. On d 21, the mRNA expression of *IL-1β*, *IL-8*, *interferon gamma* (*IFN-γ*), *tumor necrosis factor alpha* (*TNF-α*), and *IL-10* in the spleen and jejunum and *mucin 2* (*MUC2*), *claudin 1* (*CLDN1*), *occludin* (*OCLN*) and *zonula occludens 1* (*ZO-1*) in the jejunum were measured by quantitative real-time PCR (qRT-PCR) analysis. Gene specific primer sequences are shown in Table 2. The qRT-PCR assay was performed using a 7500-fluorescence detection system (Applied Biosystems, Foster City, California) and a commercial SYBR-Green PCR kit (Takara Bio Inc., Ostu, Japan). The following thermal cycler conditions were used: 95 °C for 30 s, 40 cycles of 95 °C for 5 s and 60 °C for 34 s. At the end of each run, melting curve analysis and subsequent agarose gel electrophoresis of the PCR products were carried out to confirm the amplification specificity. The *β-actin* was used as the housekeeping gene, and the data of relative gene expression were analyzed using the 2^−ΔΔCt^ method as previously described (Livak and Schmittgen, [14]). The amplifying efficiency of the qRT-PCR primers for each target gene is between 90% and 110%.

**Table 2:** Quantitative real-time PCR primer sequences

Gene name	Forward primer sequence (5′ to 3′)	Reverse primer sequence (5′ to 3′)	GenBank accession number
*β-actin*	GAGAAATTGTGCGTGACATCA	CCTGAACCTCTCATTGCCA	L08165
*IL-1β*	ACTGGGCATCAAGGGCTA	GGTAGAAGATGAAGCGGGTC	NM_204524
*IL-8*	ATGAACGGCAAGCTTGGAGCTG	TCCAAGCACACCTCTCTTCCATCC	AJ009800
*IFN-γ*	AGCTGACGGTGGACCTATTATT	GGCTTTGCGCTGGATTC	NM_205149.1
*TNF-α*	GAGCGTTGACTTGGCTGTC	AAGCAACAACCAGCTATGCAC	NM_204267
*IL-10*	CGGGAGCTGAGGGTGAA	GTGAAGAAGCGGTGACAGC	EF554720.1
*MUC2*	TTCATGATGCCTGCTCTTGTG	CCTGAGCCTTGGTACATTCTTGT	XM_421035
*CLDN1*	CATACTCCTGGGTCTGGTTGGT	GACAGCCATCCGCATCTTCT	AY750897.1
*OCLN*	ACGGCAGCACCTACCTCAA	GGGCGAAGAAGCAGATGAG	GI:464,148
*ZO-1*	CTTCAGGTGTTTCTCTTCCTCCTC	CTGTGGTTTCATGGCTGGATC	XM_413773

### Ileal and cecal microbiota enumeration

The populations of *C. perfringens*, *Escherichia* subgroup, and *Lactobacillus* subgroup species in the digesta were detected by absolute qRT-PCR, as described previously [15, 16], with some modification. Briefly, genomic DNA was isolated from about 200 mg of digesta from the ileum and caecum using a QIAamp DNA Stool Mini Kit (Qiagen Inc., Valencia, CA). Extracted DNA was stored at − 70 °C until analysis. Standard curves for qRT-PCR were prepared by normal PCR amplification using DNA extracted from pure bacterial cultures to produce a high concentration of the target DNA. Competent *Escherichia coli* DH5α (Takara Bio Inc., Japan) were used to generate plasmid standards. The PCR products were purified using a PCR purification kit (Biomed Gene Technologies, Beijing, China), and then cloned into pCR2.1 using a TA cloning kit (Invitrogen Corporation, Carlsbad, CA), as per the manufacturer’s protocol. Purified insert-containing plasmids were quantified using a Nanodrop ND-1000 spectrophotometer (Thermo Fisher Scientific Inc., Waltham, MA), and the number of target gene copies was calculated using the following formula, as described previously [17]:

$$ \mathrm{DNA}\kern0.5em \left(\mathrm{copy}\right)\kern0.5em =\kern0.5em \frac{6.02\kern0.5em \times \kern0.5em {10}^{23\kern0.5em }\left(\mathrm{copy}/\mathrm{mol}\right)\times \mathrm{DNA}\kern0.5em \mathrm{amount}\kern0.5em \left(\mathrm{g}\right)}{\mathrm{DNA}\kern0.5em \mathrm{length}\kern0.5em \left(\mathrm{dp}\right)\kern0.5em \times \kern0.5em 660\kern0.5em \left(\mathrm{g}/\mathrm{mol}/\mathrm{dp}\right)} $$

Standard curve was constructed by the ten-fold serial dilutions of plasmid DNA. Genomic DNA from ileal and cecal samples was used as a template for absolute qRT-PCR using a 7500 fluorescence detection system (Applied Biosystems, Foster City, CA) according to optimized PCR protocols (SYBR-*Premix Ex Taq*, Takara Bio Inc., Japan). The qRT-PCR primers were the same as used for normal PCR amplification (Table 3). The data were presented as log_10_ gene copies/g of intestinal digesta.

**Table 3:** The sequence of 16S rRNA quantitative real-time PCR primers used to quantify intestinal bacteria

Target	Primer sequence (5′→3′)^b^	Amplicon size, bp	Reference
*C. perfringens*	F: AAAGATGGCATCATCATTCAAC R: TACCGTCATTATCTTCCCCAAA	279	[40]
*Escherichia* subgroup^a^	F: GTTAATACCTTTGCTCATTGA R: ACCAGGGTATCTAATCCTGT	340	[41]
*Lactobacillus* subgroup	F: AGCAGTAGGGAATCTTCCA R: CACCGCTACACATGGAG	341	[15]

^a^The targeted *Escherichia* subgroup contained *E. coli*, *Hafnia alvei* and *Shigella* species

^b^*F* forward, *R* reverse

### Statistical analysis

All data were analyzed with SPSS version 20.0 (SPSS Inc., Chicago, IL). A significance level of 0.05 was used. The data of mortality and intestinal lesion score were analyzed by one-way ANOVA, and were subjected to grouped table of the GraphPad Prism 5 (GraphPad Software, Inc., CA, USA). Results in figures were represented as mean ± SE of 7 replicates, with 11 birds per replicate. Other data were analyzed by two-factorial analysis of variance to examine the main effects of probiotic and challenge, and their interaction using general linear model procedure SPSS 20.0. When a significant interaction between the main effects was observed, Duncan’s multiple comparison was used to compare the differences among the four groups. Results in the tables were given as the mean and pooled SEM.

## Results

### Growth performance and mortality

**Fig. 1:**
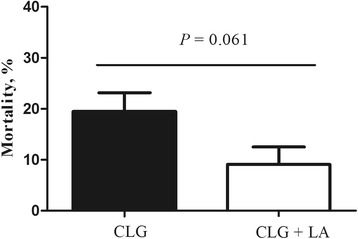
The mortality rates of treatments of *Clostridium perfringens* only challenge group (CLG) and *C. perfringens* challenge group supplemented with *Lactobacillus acidophilus* (CLG + LA) during d 14–21

The growth performance of broiler chickens was not significantly affected by dietary *L. acidophilus* addition during the d 0–14 (*P* > 0.05) (data not shown). However, as shown in Table 4, *C. perfringens* challenge decreased the ADG (*P* < 0.05) and increased the FCR (*P* < 0.05) of broilers during d 14–21. An interaction between the effects *C. perfringens* and *L. acidophilus* treatment on BW of broilers was observed on d 21 (*P* < 0.05), and *L. acidophilus* treatment significantly increased the BW of broilers when challenged (*P* < 0.05). No deaths were observed in non-challenged broilers (CTL and LA groups) during the experimental period, while *C. perfringens* challenge significantly increased the mortality rate (*P* < 0.05) of broilers from d 14 to 21, and *L. acidophilus* addition tended to decrease the mortality rate (*P* = 0.061) (Fig. 1). The growth performance of broilers during d 21–28 was not significantly affected by *C. perfringens* challenge or *L. acidophilus* supplementation (*P* > 0.05, data not shown).

**Table 4:** The growth performance of broilers during d 14–21

Item	BW at d 21, g	ADFI, g/d	ADG, g/d	FCR
CTL	750.39^a^	80.48	57.27	1.41
LA	742.38^ab^	79.26	55.58	1.43
CLG	716.52^b^	77.14	52.82	1.46
CLG + LA	751.72^a^	79.19	54.64	1.46
SEM	5.539	0.511	0.572	0.010
Main effects				
*C. perfringens* challenge				
Negative	746.38	79.87	56.43	1.42
Positive	734.12	78.46	53.73	1.46
*L. acidophilus*				
No addition	733.45	78.81	55.05	1.44
Addition	747.05	79.52	55.11	1.45
*P*-value				
*C. perfringens* challenge	0.196	0.156	0.014	0.023
*L. acidophilus*	0.242	0.465	0.949	0.589
*C. perfringens* challenge × *L. acidophilus*	0.045	0.055	0.097	0.589

^a,b^Means in the same column with different superscripts differ (*P* < 0.05)

### Intestinal lesion score

**Fig. 2:**
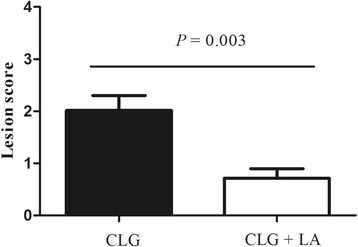
The intestinal lesion score of broilers in *Clostridium perfringens* only challenge group (CLG) and the *C. perfringens* challenge group supplemented with *Lactobacillus acidophilus* (CLG + LA) on d 21

In this study, no intestinal lesions were observed in the non-challenged broiler chickens. Most *C. perfringens-*challenged birds exhibited congested mucosa and focal haemorrhagic lesions in the jejunum and ballooning in the small intestine was observed in some challenged birds. However, *L. acidophilus* supplementation in the diet significantly decreased the intestinal lesion score (*P* < 0.05) of challenged birds (Fig. 2).

### Intestinal histomorphology

*Clostridium perfringens* challenge significantly decreased the villus height (*P* < 0.05) and the ratio of villus height to crypt depth ratio (*P* < 0.05), and tended to increase the crypt depth (*P* = 0.059, Table 5). *L. acidophilus* treatment tended to increase the villus height (*P* = 0.057) and significantly increased crypt depth (*P* < 0.05) in the both non-challenged and challenged birds.

**Table 5:** The jejunal histomorphology of broilers on d 21

Item	Villus height, μm	Crypt depth, μm	Villus height: Crypt depth
CTL	1055.81	129.02	8.13
LA	1160.26	169.4	6.87
CLG	875.04	161.96	5.64
CLG + LA	1034.86	180.23	5.84
SEM	37.187	6.475	0.289
Main effects			
*C. perfringens* challenge			
Negative	1108.03	149.21	7.50
Positive	954.95	171.10	5.74
*L. acidophilus*			
No addition	965.43	145.49	6.89
Addition	1097.56	174.82	6.36
*P*-value			
*C. perfringens* challenge	0.030	0.059	0.001
*L. acidophilus*	0.057	0.014	0.238
*C. perfringens* challenge × *L. acidophilus*	0.677	0.325	0.111

### Serum endotoxin content

The serum endotoxin was measured to estimate intestinal barrier integrity and function (Table 6). *Clostridium perfringens* challenge and *L. acidophilus* treatment had interactive effects on the levels of serum endotoxin (*P* < 0.05). The serum endotoxin content was the highest in the birds of CLG group, and was the lowest in birds of LA group. *L. acidophilus* supplementation significantly decreased the serum endotoxin content (*P* < 0.05) indespite of *C. perfringens* challenge.

**Table 6:** The endotoxin content in the serum of broilers at d 21

Item	Endotoxin, EU/mL
CTL	0.22^c^
LA	0.18^d^
CLG	0.39^a^
CLG + LA	0.27^b^
SEM	0.015
Main effects	
*C. perfringens* challenge	
Negative	0.20
Positive	0.33
*L. acidophilus*	
No addition	0.30
Addition	0.23
*P*-value	
*C. perfringens* challenge	< 0.001
*L. acidophilus*	< 0.001
*C. perfringens* challenge × *L. acidophilus*	< 0.001

^a-d^Means in the same column with different superscripts differ (*P* < 0.05)

### *MUC2*, *CLDN1*, *OCLN and ZO-1* mRNA expression

As shown in Table 7, *C. perfringens* infection significantly decreased the *OCLN* mRNA expression (*P* < 0.05), while *L. acidophilus* treatment tended to decrease *MUC2* mRNA expression in the jejunum (*P* = 0.082). The relative mRNA levels of *CLDN1* and *ZO-1* expression in the jejunum were not significantly affected by *C. perfringens* infection or *L. acidophilus* treatment (*P* > 0.05).

**Table 7:** The mRNA expression levels of *MUC2*, *CL-1*, *OCLN*, and *ZO-1* in the jejunum of broilers at d 21 (log_2_ relative)

Item	*MUC2*	*CLDN1*	*OCLN*	*ZO-1*
CTL	1.01	1.01	1.05	1.01
LA	0.87	1.07	0.99	0.93
CLG	0.98	1.16	0.84	1.06
CLG + LA	0.79	1.11	0.82	0.92
SEM	0.046	0.062	0.037	0.040
Main effects				
*C. perfringens* challenge				
Negative	0.94	1.04	1.02	0.97
Positive	0.88	1.14	0.83	0.99
*L. acidophilus*				
No addition	0.99	1.09	0.95	1.04
Addition	0.83	1.09	0.90	0.93
*P*-value				
*C. perfringens* challenge	0.518	0.474	0.010	0.850
*L. acidophilus*	0.082	0.983	0.506	0.206
*C. perfringens* challenge × *L. acidophilus*	0.781	0.670	0.767	0.732

### Cytokine mRNA expression

The expression of *IL-1 β*, *IFN-γ*, and *TNF-α* in the spleen was significantly (*P* < 0.05) up-regulated by *C. perfringens* challenge (Table 8), while *L. acidophilus* treatment alone significantly (*P* < 0.05) down-regulated the expression of *IL-1 β* and *TNF-α*. Interestingly, interactive effects on the *IL-8* mRNA expression (*P* < 0.05) in the spleen were observed between *C. perfringens* challenge and *L. acidophilus* treatment. *IL-8* mRNA expression in the spleen of broilers in CLG treatment was significantly higher than that in non-challenged birds (*P* < 0.05). However, the *IL-8* mRNA expression in the spleen was significantly (*P* < 0.05) decreased in the challenged broilers fed *L. acidophilus*-supplemented diet.

**Table 8:** Cytokine mRNA expression levels in the spleen of broilers at d 21 (log_2_ relative)

Item	*IL-1 β*	*IL-8*	*IFN-γ*	*TNF-α*	*IL-10*
CTL	1.03	1.02^b^	1.04	1.00	1.15
LA	0.90	1.09^b^	0.98	0.92	1.32
CLG	1.29	1.30^a^	1.25	1.22	0.88
CLG + LA	1.08	1.03^b^	1.13	0.94	1.38
SEM	0.046	0.039	0.043	0.034	0.118
Main effects					
*C. perfringens* challenge					
Negative	0.96	1.06	1.01	0.96	1.23
Positive	1.18	1.17	1.19	1.08	1.13
*L. acidophilus*					
No addition	1.16	1.16	1.14	1.11	1.02
Addition	0.99	1.06	1.06	0.93	1.35
*P*-value					
*C. perfringens* challenge	0.009	0.153	0.039	0.034	0.665
*L. acidophilus*	0.043	0.118	0.302	0.002	0.180
*C. perfringens* challenge × *L. acidophilus*	0.619	0.020	0.758	0.079	0.496

^a,b^Means in the same column with different superscripts differ (*P* < 0.05)

As shown in Table 9, *C. perfringens* challenge increased the mRNA expression of *IL-1 β* (*P* < 0.05) in the jejunum. Conversely, *L. acidophilus* treatment significantly decreased the expression of *IL-1 β* (*P* < 0.05), and tended to decrease *TNF-α* expression in the jejunum of broilers at d 21 (*P* = 0.074). Interactive effects on the expression of *IL-8* (*P* < 0.05), *IFN-γ* (*P* < 0.05), and *IL-10* (*P* < 0.05) in the jejunum were observed between *C. perfringens* challenge and *L. acidophilus* treatment. Furthermore, *L. acidophilus* treatment decreased the expression of *IL-8* (*P* < 0.05), *IFN-γ* (*P* < 0.05), and *IL-10* (*P* < 0.05) in the jejunum of challenged broilers.

**Table 9:** Cytokine mRNA expression levels in the jejunum of broilers at d 21 (log_2_ relative)

Item	*IL-1 β*	*IL-8*	*IFN-γ*	*TNF-α*	*IL-10*
CTL	1.14	1.43^b^	1.05^a^	1.05	1.30^a^
LA	1.06	0.75^b^	1.46^a^	0.89	2.43^a^
CLG	2.73	3.55^a^	1.49^a^	1.02	2.58^a^
CLG + LA	1.36	0.79^b^	0.92^b^	0.80	1.19^b^
SEM	0.205	0.318	0.101	0.052	0.243
Main effects					
*C. perfringens* challenge					
Negative	1.10	1.09	1.25	0.97	1.86
Positive	2.05	2.17	1.20	0.91	1.89
*L. acidophilus*					
No addition	1.94	2.49	1.27	1.04	1.94
Addition	1.21	0.77	1.19	0.85	1.81
*P*-value					
*C. perfringens* challenge	0.009	0.037	0.789	0.570	0.961
*L. acidophilus*	0.041	0.002	0.677	0.074	0.775
*C. perfringens* challenge × *L. acidophilus*	0.066	0.043	0.016	0.747	0.009

^a,b^Means in the same column with different superscripts differ (*P* < 0.05)

### Enumerations of ileal and cecal microbiota

The populations of *C. perfringens* (*P* < 0.05), *Lactobacillus* (*P* < 0.05), and *Escherichia* (*P* < 0.05) in the ileum and populations of *C. perfringens* (*P* < 0.05) and *Escherichia* (*P* < 0.05) in the cecum were significantly increased by *C. perfringens* challenge (Table 10), and *Lactobacillus* number in the cecum of broilers at d 21 was tended to be increased by challenge (*P* = 0.098). *L. acidophilus* supplementation into the diet significantly (*P* < 0.05) increased the *Lactobacillus* population in the both ileum and cecum, and significantly (*P* < 0.05) decreased the *Escherichia* population in the ileum of broilers at d 21.

**Table 10:** The quantitation of intestinal microbiota of broilers on d 21

Item^a^	Ileum			Cecum		
	*C. perfringens*	*Lactobacillus*	*Escherichia*	*C. perfringens*	*Lactobacillus*	*Escherichia*
CTL	1.16	6.25	6.19	4.64	6.61	8.93
LA	2.32	7.17	5.83	5.29	7.28	9.00
CLG	3.46	7.15	6.83	6.42	7.11	9.56
CLG + LA	3.62	7.55	6.13	6.08	7.29	9.35
SEM	0.316	0.136	0.125	0.251	0.089	0.091
Main effects						
*C. perfringens* challenge						
Negative	1.74	6.71	6.01	4.97	6.94	8.97
Positive	3.54	7.35	6.48	6.25	7.20	9.45
*L. acidophilus*						
No addition	2.31	6.70	6.51	5.53	6.87	9.24
Addition	2.97	7.36	5.98	5.69	7.29	9.18
*P*-value						
*C. perfringens* challenge	0.003	0.006	0.025	0.009	0.098	0.006
*L. acidophilus*	0.225	0.005	0.046	0.733	0.010	0.698
*C. perfringens* challenge × *L. acidophilus*	0.360	0.232	0.433	0.284	0.115	0.393

^a^The results are expressed as log_10_ (copies/g digesta)

## Discussion

The current study highlighted the significance of *C. perfringens* infection in thepoultry production. The mortality rate of *C. perfringens*-challenged birds was the highest out of all groups examined in this study, but was lower than the 36% mortality rate reported in a previous clinical NE study [18], which might indicate the strain specificity of *C. perfringens*. Pathogen challenge also increased the intestinal lesion score and decreased the intestinal villus height and the ration of villus height to crypt depth ratio in the present study. Intestinal NE lesions and mucosal atrophy greatly compromises epithelial permeability and mucosal barrier function [19], resulting in bacteria translocation to the liver, spleen, and blood [13]. This is reflected in increased levels of blood endotoxin produced by Gram-negative bacteria. Accordingly, we observed that the serum endotoxin content of broilers in the infected groups was higher than that in the uninfected groups.

Lactobacilli can inhibit pathogen growth and enhance poultry productivity. However, there are few reports on the effects of lactobacilli on NE-infected broilers. Geier et al. [18] reported that *L. johnsonii* appeared to reduce intestinal lesion score and the ratio of feed to weightgain, but did not decrease the mortality of NE-infected broilers. *L. fermentum* 1.2029 reduced the NE lesion severity and attenuated intestinal damage caused by *C. perfringens* challenge [20]. In the current study, *L. acidophilus* supplementation in the diet improved growth performance, reduced the mortality rates, and attenuated cellular damage of challenged broilers. Italso increased the jejunal crypt depth, which might aid the intestinal renewal and the recovery of NE-infected broiler chickens.

Tight junctions are the most important components of the intestinal epithelial cell barrier, which protects the host from intestinal pathogens and prevents macromolecular transmission [21]. Tight junctions are formed by several types of proteins, including OCLN, claudins, junctional adhension molecules, and zonula occludens (ZO) proteins [22]. To date, only *claudin-1*, *claudin-2*, *claudin-3*, *claudin-5*, *claudin-16*, *ZO-1*, *ZO-2*, and *OCLN* have been reported in poultry [23–26]. In the present study, *C. perfringens* challenge decreased the *OCLN* mRNA expression, but did not significantly affect the expression of *CLDN1* or *ZO-1* in the jejunum. This finding was consistent with a previous report [13], which demonstrated that *OCLN* mRNA expression in the jejunum and ileum was down-regulated by *C. perfringens* infection. Du et al. [27] also reported that the *CLDN1* expression in the ileum was decreased by *C. perfringens* challenge. However, in the current study, *L. acidophilus* treatment did not show significant effect on the expression of *CLDN1*, *OCLN* or *ZO-1*, but did decrease *MUC2* mRNA expression. The specific strain of *L. acidophilus* used in this study might not benefit the host by strengthening the tight junctions.

Many types of bacteria have been shown to co-exist in case of NE, and one of the largest populations is *E. coli* [28], although *Lactobacillus* species arepresent as well [13, 29]. In the current study, *C. perfringens* challenge increased the populations of *C. perfringens* and *Escherichia* in the ileum and cecum, and also increased ileal *Lactobacillus* counts*,* which is consistent with previous reports [13, 16]. Lactobacilli can prevent the growth of *C. perfringens* in vitro [30]. It was also demonstrated that some *Lactobacillus* strains have demonstrated the capacity to prevent *C. perfringens* colonization and NE outbreak in vivo. Addition of *Lactobacillus* sp. NO. I-2673 to drinking water decreased the cecal *C. perfringens* populations of healthy chickens [31], while La Ragione et al. [32] reported that *L. johnsonii* FI9785 prevented *C. perfringens* colonization in pathogen-free broilers. However, Geier et al. [18] observed that *L. johnsonii* did not exhibit any an-ticlostridial properties in vivo. The differences among these reports may indicate strain specificity of *L. johnsonii* fighting against *C. perfringens*. In this study, supplementation of *L. acidophilus* in the diet increased *Lactobacillus* numbers in the ileum and cecum, and decreased the ileal *Escherichia* counts, which might help counteract the increased serum endotoxin content induced by *C. perfringens* challenge. However, *L. acidophilus* treatment did not significantly affect the intestinal *C. perfringens* counts in the current study. In contrast, Fukata et al. [33] reported that *L. acidophilus* could inhibit the toxins production of *C. perfringens*, which might help explain the beneficial effect after probiotic addition.

The host immune responses are triggered after birds are infected by a pathogen. If the pathogen-induced inflammatory responses get out of control, tissue damage occurs, accompanied by suboptimal growth performance, which was also observed in the current study. Previous studies have demonstrated that theexpression of *IFN-γ*, *IL-1β*, *IL-4*, and *IL-10* was increased in the intestines of NE-infected broilers compared with uninfected controls [34, 35]. However, the immune responses to *C. perfringens* infection may differ due to the varieties of broiersand vary in different immune organs. Hong et al. [36] observed that NE-infected broilers showed up-regulated transcription of the pro-inflammatory cytokine-encoding genes *IL-1β*, *IL-6*, *IL-17F*, and *TNFSF15* in the spleen and *TNFSF15* in the intestine, but the expression of *IL-17F* was only increased in the intestines of Ross chickens, and was not significantly affected in the intestines of Cobb chickens. In addition, Lee et al. [37] reported that *IL-8*, *LITAF*, *TNFSF15*, *IL-17A* and *IL-17F* transcript levels in the jejunum of broilers were up-regulated by *Eimeria maxima*/*C. perfringens* co-infection, while only *IL-17A* transcripts were increased in the spleen. In this study, the treatments affected the *IL-10* mRNA expression level in the jejunum, but not in the spleen. What’s more, the inflammatory responses seemed to be more intense in the jejunum than in the spleen, which might lead to gut damage, higher energy consumption and sub-optimal growth performance. The inconsistency of previous findings and present study might be due to differences of the NE infection models and broiler varieties.

Lactobacilli has been shown to regulate the transcript levels of cytokines. In the absence of pathogen challenge, *L. acidophilus* was effective in inducing T-helper-1 cytokines, while *L. salivarius* induced an even greater anti-inflammatory response in the chicken spleen and cecal tonsil cells [38]. Wang et al. [39] reported that novel *L. plantarum* strain P-8 increased the jejunal transcription of *IFN-γ*, *IL-12*, and *IL-4* at d 14 post treatment. Those studies demonstrated that lactobacilli could increase host immunity in the absence of pathogens. However, little was known aboutthe effects of Lactobacilli on the cytokine expression in a NE model. In the present study, *L. acidophilus* decreased the transcription of *IL-1β* and *TNF-α* in the spleen and jejunum of broilers, irrespective of *C. perfringens* challenge. In addition, the decreases of *IL-8* expression were observed in the spleen, while the expression levels of *IL-8*, *IFN-γ*, and *IL-10* were decreased in the jejunum of NE-infected broilers fed *L. acidophilus* supplemented diet compared with those infected broilers fed control diet. Overall, lactobacilli may exhibit different regulatory functions when hosts were under different conditions. Thus, the decreased cytokine levels may be associated with the protective effect of *L. acidophilus* treatment onthe gut health of NE-infected broilers. *L. acidophilus* treatment ameliorated the inflammation, and therefore reduced the consumption of energy and improved gut health, as evidenced by the decreased lesion scores and increased villus height.

## Conclusions

Dietary supplementation with *L. acidophilus *could alleviate the inflammation and intestinal impairment, improve intestinal morphology and barrier integrity, and modulate the intestinal microflora. Consequently, *L. acidophilus* addition benefited the intestinal health, and decreased the mortality of broilers challenged with *C. perfringens*.

## Abbreviations

ADFI: Average daily feed intake
ADG: Average daily gain
BW: Body weight
*C. perfringens*: *Clostridium perfringens*
CLDN1: Claudin 1
FCR: Feed conversation ratio
FI: Feed intake
IFN-γ: Interferon gamma
*L. acidophilus*: *Lactobacillus acidophilus*
MUC2: Mucin 2
NE: Necrotic enteritis
OCLN: Occludin
TNF-α: Tumor necrosis factor alpha
ZO-1: Zonula occludens 1
